# The application of MTA in single visit apexification treatment: A prospective study

**DOI:** 10.6026/973206300214741

**Published:** 2025-12-15

**Authors:** S.K Mahboob Rahaman, Sagolsem Chandarani, Anuradha Mukherjee, Thara Elizabeth Jes, Prantika Mandal, Abhijeet Buragohain

**Affiliations:** 1 Department of Conservative Dentistry and Endodontics, North Bengal Dental College and Hospital, Darjeeling, West Bengal, India; 2 Department of Conservative Dentistry and Endodontics, Regional Institute of Medical Sciences, Imphal, Manipur, India; 3 Department of Conservative Dentistry and Endodontics, Hazaribag College of Dental Sciences and Hospital, Hazaribag Jharkhand, India; 4 Department of Dentistry, Assam Medical College and Hospital, Dibrugarh, Assam, India

**Keywords:** Apexification, endodontic procedure, immature teeth, MTA, periapical inflammation

## Abstract

Apexification is a treatment procedure used to induce a calcified barrier at the apex of an immature tooth with an open root canal, often as a result of pulpal necrosis or trauma. Hence, this prospective study evaluates the clinical efficacy of mineral trioxide aggregate (MTA) in achieving apexification in a cohort of 85 patients with immature, non-vital teeth. A total of 85 patients with immature permanent teeth requiring apexification were enrolled in this prospective clinical study. Each patient underwent a one-visit apexification procedure using MTA as the apical barrier material. Radiographic analysis showed the formation of a calcified barrier in most cases. Thus, one-visit apexification using MTA is an effective and efficient technique for managing immature, non-vital teeth.

## Background:

Apexification is a vital endodontic procedure used to treat immature, non-vital permanent teeth with open apices. The primary objective of apexification is to promote the formation of a calcified apical barrier, allowing the canal to be filled and sealed to prevent reinfection. Previously, apexification had been performed with calcium hydroxide which is less inferior to mineral trioxide aggregate (MTA) [1]. More recently however, a material identified as Mineral Trioxide Aggregate or MTA has come to light as the most probable substitute for apexification. MTA is a biocompatible material with radiopacity, excellent sealing property, antimicrobial activity and the potential to stimulate hard tissue regeneration. While calcium hydroxide permits apexification, MTA allows one visit apexification which is more time effective and less costly in terms of patient visits but with higher success rates [2]. Application of MTA has already been implemented in endodontics more frequently because not only does it make the procedure easier, but it also provides better result in terms of formation of apical barrier and definitiveness of tooth [3]. Due to the above-stated properties, MTA can help in the formation of osteodentin-like tissue which helps in the formation of a calcified layer in the apex [4]. The other advantage of the material is that it kills the remaining bacteria in the canal system leading to faster healing of the periapical tissues. This one-visit technique decreases patient discomfort, compliance issues and the therapeutic load on the patient and clinician [5]. The old approach to apexification using calcium hydroxide is that it is time-consuming, Faber visits several appointments, apexification cannot predict the formation of a barrier and treatment failure is possible if the layer does not form. Compared to that, MTA apexification enables one-visit treatment and the different comparisons demonstrated better clinical efficacy. MTA is thus an important material to manage immature teeth with open apices because it can induce hard tissue formation in a single visit [6]. Therefore, it is of interest to evaluate the efficacy of one-visit apexification using MTA in a cohort of 85 patients with immature, non-vital teeth.

## Methodology:

A prospective clinical study was conducted. A total of 85 patients with immature, non-vital teeth requiring apexification were recruited. Each patient underwent a one-visit apexification procedure using MTA as the apical barrier material.

## Inclusion criteria:

[1] Patients with immature, non-vital permanent teeth.

[2] Teeth with open apices requiring apexification.

[3] No history of prior endodontic treatment on the affected tooth.

## Exclusion criteria:

[1] Teeth with vertical root fractures.

[2] Patients with systemic conditions that affect bone healing (e.g., diabetes, osteoporosis).

[3] Patients with active infection requiring additional treatment.

## Data collection:

Data for this study were collected from 85 patients with immature, non-vital permanent teeth requiring apexification. Each patient underwent a one-visit apexification procedure using MTA as the apical barrier material. Clinical findings were tabulated at baseline, 1 month, 3 months and 6 months post-treatment to determine the development of calcified apical barrier. To examine apical barrier formation, radiographic analysis was also employed and clinical examination was performed to detect any sign that indicated infection or inflammation. Other source of data includes age; gender and type of tooth were also recorded as variables. All the procedures were done by endodontist only making sure that the same was being done by one person all the time hence same on the use of material.

## Data analysis:

Data were analyzed using SPSS v29. Mean and standard deviation (S.D.) for quantitative data were calculated. A post-stratification Chi-square test was applied, with a p-value <0.05 considered statistically significant.

## Results:

Table 1 (see PDF), Figure 2 (see PDF) shows the success rate of MTA apexification increased over time. At 1 month, 84.7% of cases demonstrated evidence of apical barrier formation, which increased to 94.1% by 6 months, indicating that MTA provides sustained improvements over time. (Table 2 - see PDF) demonstrates the progression of apical barrier formation over the follow-up period. At 1 month, 60 cases showed complete barrier formation, which increased to 80 cases by 6 months, indicating the consistent efficacy of MTA in promoting hard tissue formation. The number of cases with no barrier formation dropped to zero by the final follow-up, showcasing the high success rate of this technique. Pain decreased from 40 at baseline to just 2 after 6 months. Sensitivity and swelling also reduced drastically, with sensitivity dropping from 35 to 1 and swelling from 25 to 0. Additionally, the percentage of individuals experiencing no symptoms increased substantially, rising from 0 at baseline to 82 after 6 months (Table 3 - see PDF, Figure 1 - see PDF). The treatment success rates for different tooth types show high effectiveness. Incisors had the highest success rate at 96.7%, followed by canines at 95.0%, premolars at 92.0% and molars at 90.0%. Overall, the success rates remain consistently high across all tooth types. The majority of patients reported high satisfaction, with 82.3% being very satisfied. 11.8% were somewhat satisfied, while 5.9% remained neutral. No patients expressed dissatisfaction, indicating a highly positive response overall (Figure 3 - see PDF). The success rates varied with the apical diameter. Treatments with an apical diameter of less than 1 mm had a perfect success rate of 100%. Those in the 1-2 mm range had a success rate of 95.0%, while treatments with an apical diameter greater than 2 mm had a slightly lower success rate of 86.7% (Table 4 - see PDF, Figure 4 - see PDF).

## Discussion:

The results of this study provide compelling evidence for the effectiveness of one-visit apexification using MTA. Reporting 6 months success rate of 94.1%, it revealed that MTA has the potential to form an apical barrier in a single visit. In comparison with the conventional multiple appointment calcium hydroxide apexification, MTA has the advantages of short treatment time, increased patient acceptance and better apical barrier formation [7]. A sound achievement of this study is that at the end of six months, a variable degree of calcification of the apical barrier had formed in most cases as evidenced by radiographic follow- up. These features contribute to the formation of hard tissue due to the effectiveness of MTA in comparison with other materials for stimulated differentiation of dental pulp stem cells and deposition of dentin-like material. This is made possible through the bio-activity of MTA, which dissolves in water to release calcium ions that precipitate as calcium hydroxide against the material-tissue boundary. This forms a calcium layer that sustains the hard tissue formation and thus the development of a calcified barrier at the apex [8, 9]. The second major finding of this work is the increased average score, indicating a reduction in clinic symptoms in patients under the surgeon's care. Since the apexification protocol of MTA, most of the patients reported decreased levels of pain, sensitivity and swelling. More than 82% of patients maintained to be "very satisfied" with the single-visit approach by 6 months, identifying it as just about the greatest asset of the method. MTA has the advantage of restoring the function of the tooth immediately and does not need, as does calcium hydroxide, several appointments and a long-time exposure to the tooth. Furthermore, the high satisfaction with the treatment offered is in concordance with past research that has pointed to the role of efficient treatment pathways that characterize contemporary endodontic practice [10]. This study also assessed the effect of tooth type and size of the apical foramen opening on treatment results. Analyzing the data and results it is possible to state that the success rate of smaller apical diameters (less than 1mm) was 100% whereas for larger apical diameters (more than 2mm) the success rate was 86.7 %. These results are in line with earlier investigations showing that in the cases of smaller obturating cone apical diameters the support for MTA and hard tissues was more favorable [11]. Maxillary central incisors had the highest success rates since their root canal morphology was less complex than that of other teeth; mandibular molars and premolars had slightly lower success rates. This may be attributed to the increased intricacies of the root canal system of the posterior teeth, in so far as the placement and condensation of MTA [12]. The literature review revealed that MTA possesses the property to stimulate hard tissue formation and apical barrier formation. Some authors have reported MTA's biocompatibility and bioactivity about cytokines and growth factors that induce the differentiation of osteoblasts and cementoblasts. The liberization of calcium hydroxide of MTA creates an environment that favors the precipitation of calcium phosphate and hence formation of the mineralized matrix. This process closely resembles the formation of dentin and cementum and, therefore, MTA is the material of choice for one-visit apexification [13, 14]. The effect of these findings has implications for the practice of endodontics. The earlier practice of using calcium hydroxide involved several visits besides having the probability of treatment failure if the apical barrier is not formed. In this regard, the fact that MTA can stimulate the formation of the barrier within one visit gets rid of lengthy treatment processes. Furthermore, since it can harden in a wet condition and can act as an antimicrobial barrier, it should be applied to cases with an active infection or periapical lesions [15, 16]. This study's findings align with previous research supporting MTA as the gold standard for apexification [17, 18]. The ability to complete the treatment in a single visit improves patient compliance, minimizes clinical visits and enhances overall patient satisfaction [19]. Future research should focus on improving the handling characteristics of MTA, reducing its cost of production and exploring new bioactive materials that may further enhance treatment outcomes.

## Conclusion:

The effect of one-visit apexification using MTA for treating immature, non-vital teeth, achieving a 94.1% success rate at 6 months is shown. The procedure demonstrated significant improvements in apical barrier formation, pain reduction and patient satisfaction, with 82.3% of patients reporting being "very satisfied". Thus, we show the advantages of MTA over traditional multi-visit calcium hydroxide apexification, including fewer clinical visits, faster barrier formation and higher patient compliance.
